# Morphological study of pulp cavity anatomy of canine teeth in domestic cats using micro-computed tomography

**DOI:** 10.3389/fvets.2024.1373517

**Published:** 2024-03-08

**Authors:** Emilia Chrostek, Santiago Peralta, Nadine Fiani

**Affiliations:** Department of Clinical Sciences, College of Veterinary Medicine, Cornell University, Ithaca, NY, United States

**Keywords:** feline, endodontics, canine tooth, apical delta, micro-CT, pulp cavity

## Abstract

An understanding of the pulp cavity anatomy of individual teeth is essential for success during endodontic therapy. The objective of this study was to document pulp cavity anatomy and summarize numerical data of maxillary and mandibular canine teeth of domestic cats using micro-computed tomography (micro-CT). Thirty-nine canine teeth from eleven domestic cat cadaveric specimens were extracted and prepared for scanning. Segmentation of the pulp cavity was performed using the Avizo (v2022.2) software package. The morphological features of the pulp cavity including overall shape, configuration, presence of apical deltas and lateral canals was recorded. A quantitative analysis was performed on thirty-one teeth to explore associations between pulp cavity volume and length, apical delta length, maximum apical delta foramina number and cusp-to-tip length using a linear mixed model. Correlation between pertinent continuous variables was assessed using a Pearson’s correlation test. Most pulp cavities exhibited varying curvature and ranged from a cylindrical configuration in the coronal third to an ovoid configuration in the middle to apical third. A ribbon-like flattened canal was observed in 6/31 teeth (19%). All canine teeth depicted an apical delta with various configurations except for two teeth that showed a single canal exiting at the apex. In 15/31 teeth (48%), the primary root canal within the apical delta could be clearly identified and in 16/31 (52%) the primary root canal was indiscernible. The results showed that the pulp cavities of maxillary canine teeth were significantly larger and longer and the cusp-to-tip length was longer, when compared to mandibular teeth. The apical delta length was negatively correlated to the volume of the pulp cavity. No specimens depicted lateral canals. This study revealed that the anatomy of the canine tooth pulp cavity in cats can vary considerably and should be a consideration when performing thorough debridement, shaping and obturation of the endodontic system.

## Introduction

An in-depth knowledge of the pulp cavity morphology of a tooth is essential when considering endodontic treatment. The unique 3D internal anatomy of a tooth presents challenges when attempting successful access, disinfection, shaping, and obturation of the endodontic system. The anatomy of a pulp cavity can be separated into two parts: the pulp chamber located in the anatomic crown and the root canal located in the anatomic root (1, 2). Accessory canals can extend from the pulp space to the periodontium and usually contain minor vessels and connective tissue. At the root canal apex, various morphological configurations have been observed (3–7). An apical foramen is characterized by a rounded edge that differentiates the end of the cemental canal from the outer surface of the root (1). In contrast, an apical delta is described as multiple accessory canals that branch out from the main canal at or near the root apex (1).

The apical root canal anatomy in cats has been shown to be vastly different from that in humans (5). A complex apical delta architecture with multiple foramina has been described in both dogs and cats (3–5). The most common method for examining apical deltas in veterinary studies has previously been based on tooth clearing, which inevitably results in destruction of the sample and is inaccurate for quantitative data (8–10). This method was used to show the apical anatomy of canine teeth in cats, which confirmed an apical delta rather than a primary apical foramen commonly observed in humans (5).

Most endodontic studies in veterinary medicine focus on the 2D characteristics of a 3D object and thus present limitations. Few veterinary studies have used micro-computed tomography (micro-CT) to assess the morphology of teeth (11–18). As such, the current understanding of the anatomy of the feline canine tooth pulp cavity stems primarily from the study of intraoral radiographic images. The use of micro-CT has become an increasingly important tool in the study of endodontics as it provides an accurate, non-invasive 3D *ex vivo* assessment of the root canal system (2, 17, 19–24). Root canal morphology of different teeth in human populations has been extensively studied using micro-CT and has shown considerable anatomical variation (2, 19, 20, 24–33). The first study that described the use of micro-CT for endodontics in human teeth was performed by Nielsen et al. (21). Although micro-CT has limited use for *in vivo* imaging, it is an important tool for describing the fine details of root canal anatomy.

Root canal treatment in cats is often a technically challenging procedure due to the small size of domesticated feline teeth (34, 35). As a result, the canine tooth is the most common endodontically-treated tooth in the cat. Previous studies have suggested significantly higher failure rates of endodontically treated teeth in felines as compared to canine species (35, 36). Defining the anatomical features of the pulp cavity allows the practitioner to be aware of possible challenges and allows for appropriate instrumentation (37). The importance of understanding the endodontic system anatomy has been shown in multiple studies that demonstrate that canal geometry has a greater effect on enlargement and shaping than the actual instrumentation technique (1, 20, 38, 39).

The aim of this study was to systematically document the morphological characteristics and perform a quantitative analysis of maxillary and mandibular canine teeth pulp cavities in cats using micro-CT. Furthering our understanding of the canine tooth pulp cavity anatomy in cats may lead to the development of more appropriate treatment methods to increase the success of endodontically-treated teeth.

## Materials and methods

### Specimen selection, tissue collection, and intraoral radiography

Maxillary and mandibular canine teeth were extracted from cadaveric specimens. Eleven domestic cat fresh cadaveric specimens were commercially obtained from Skulls Unlimited (Oklahoma City, OK) for this study. Specimens were of unknown age, sex, and breed. Intraoral radiographs were obtained using dedicated dental radiographic equipment and intraoral photostimulable phosphor plate (PSP) systems (CS 7600, Carestream Dental, Atlanta, GA; Scan X Duo, Air Techniques, Melville, NY). Standard maxillary and mandibular occlusal and lateral canine projections were obtained using the bisecting angle technique for each head prior to extraction. Based on oral examination and intraoral radiographic findings, canine teeth were excluded if there was evidence of tooth resorption, periodontitis, incomplete apexogenesis, evidence of endodontic disease or if there was evidence of previous endodontic treatment. These exclusion criteria were based on previously described methodology (27, 29–31). The canine teeth were then ultrasonically scaled and extracted from the head using open extraction technique performed by a board-certified veterinary dentistry specialist and a dentistry and oral surgery resident in training. Immediately after extraction, the teeth were soaked in 5.25% NaOCl for 2 h and then stored in distilled water at room temperature until analyzed, as detailed in other studies (23, 27, 30).

### Micro-CT evaluation

Extracted canine teeth were numbered in a manner to identify the cadaveric specimen they came from and whether they were maxillary or mandibular. Radiolucent hydro-plastic beads were used to fashion trays that could hold up to 6 teeth. Two to three trays of teeth were stacked at once in the micro-CT tube with the plane of the tray orientated parallel to the axis of rotation of the CT. This ensured that the long axes of the teeth were aligned parallel to the axis of rotation, resulting in a superior image quality in comparison to other orientations. The trays were secured in the micro-CT tube to ensure no movement for the duration of the scan. The teeth were scanned using a high-resolution micro-CT (Skyscan 1,276, Bruker, Germany) and scanned at an x-ray energy of 100 kV, with an aluminum and copper filter, using a slice thickness of 10 μm. Image data was exported in TIFF (Tagged Image File) format and then converted to AM (AmiraMesh) format for use in a dedicated digital imaging visualization and analysis software (Avizo v2022.2) for viewing by a dentistry and oral surgery resident in training (EC). Multiplanar reconstructions (MPR) in the transverse, sagittal and dorsal planes were utilized for analysis.

### Segmentation of the pulp cavity and 3D reconstruction

Segmentation of the pulp cavity was performed to obtain a reconstructed 3D image. All segmentation was performed using the AVIZO software package (v2022.2, http://www.fei.com/software/avizo3d/) and specific steps were carried out in the same manner for all teeth with individualized manual refinements as needed. Segmentation allows a group of voxels to be extracted from the rest of the data using intensity and density, allowing for accurate extraction of the endodontic system (40).

Segmentation of the endodontic system involved multiple steps. In the first step, the endodontic system was split into (1) “top,” which included the pulp cavity to just prior to the start of the apical delta; (2) “bottom,” which comprised of the start of the apical delta to the bottom of the longest canal (Supplementary Figure S1). Subsequently, a course outline and a fine outline was created for both the “top” and the “bottom.” Four separate materials were created to reflect this and named “top course,” “top fine,” “bottom course” and “bottom fine.” The course material was created by using the “Brush” tool followed by interpolation of the selected slices. The fine material was created by using the “Magic Wand” tool. Manual refinements of the pulp cavity were performed through the “Lasso” and “Brush” tool in the transverse, sagittal and dorsal planes to adequately account for canals in all planes, remove small spots and eliminate imaging artifacts. For the “top” and “bottom” reconstructions, the “Fill” and “Smooth” function were utilized across all slices to further refine the image. The final images of the “top” and “bottom” were combined into a new material, resulting in a complete and detailed representation of the pulp cavity structure (Supplementary Figure S1).

### Data acquisition

After segmentation of the endodontic system, data acquisition was performed by a veterinary dentist resident in-training (EC) with guidance from board-certified veterinary dentists (NF, SP). The 3D anatomy and morphological characteristics of the endodontic system of maxillary and mandibular canine teeth in cats were analyzed using a set of criteria adapted from multiple published human micro-CT endodontic morphologic studies (26, 41, 42). The shape of the pulp cavity at specific slices and examination of the images in the transverse, sagittal and dorsal planes of each tooth was recorded. A 3D reconstruction of the apical portion of the canine teeth was completed. Particular attention was paid to examining the configuration of the apex and the maximum number of foramina observed at the apex was recorded for each tooth when applicable. The presence and location of lateral and accessory canals was documented for each tooth.

A quantitative analysis of the endodontic system of the teeth was also performed on the segmented 3D pulp cavities. The measurements were obtained through the “Label Analysis” algorithm available in the Avizo software program and included total volume, total 3D area and total 3D length of the pulp cavity of each canine tooth. The total length of the apical delta was measured by counting the number of slices from the transverse section of the first branch off the main root canal to the bottom of the tooth and multiplied by 10 to account for 10 μm/slice. The final length was then converted into millimeters. For interest, the distance between the most coronal aspect of the pulp chamber to the external tip of the cusp was measured using the “Line” tool and recorded in millimeters.

### Statistical analysis

Statistical analysis of results was performed using commercially available software (JMP Pro 15, SAS Institute Inc., Cary, NC). *p*-values <0.05 were considered significant. Categorical data were described as the frequency of occurrence. Numerical data was summarized using median, interquartile range (IQR), mean, standard deviation, minimum, and maximum. Correlation between pertinent continuous variables was assessed using a Pearson’s correlation test. Numerical variable differences between maxillary and mandibular teeth were evaluated using a linear mixed model accounting for cadaver identification as a random effect.

## Results

### Quantitative analysis

**Figure 1:**
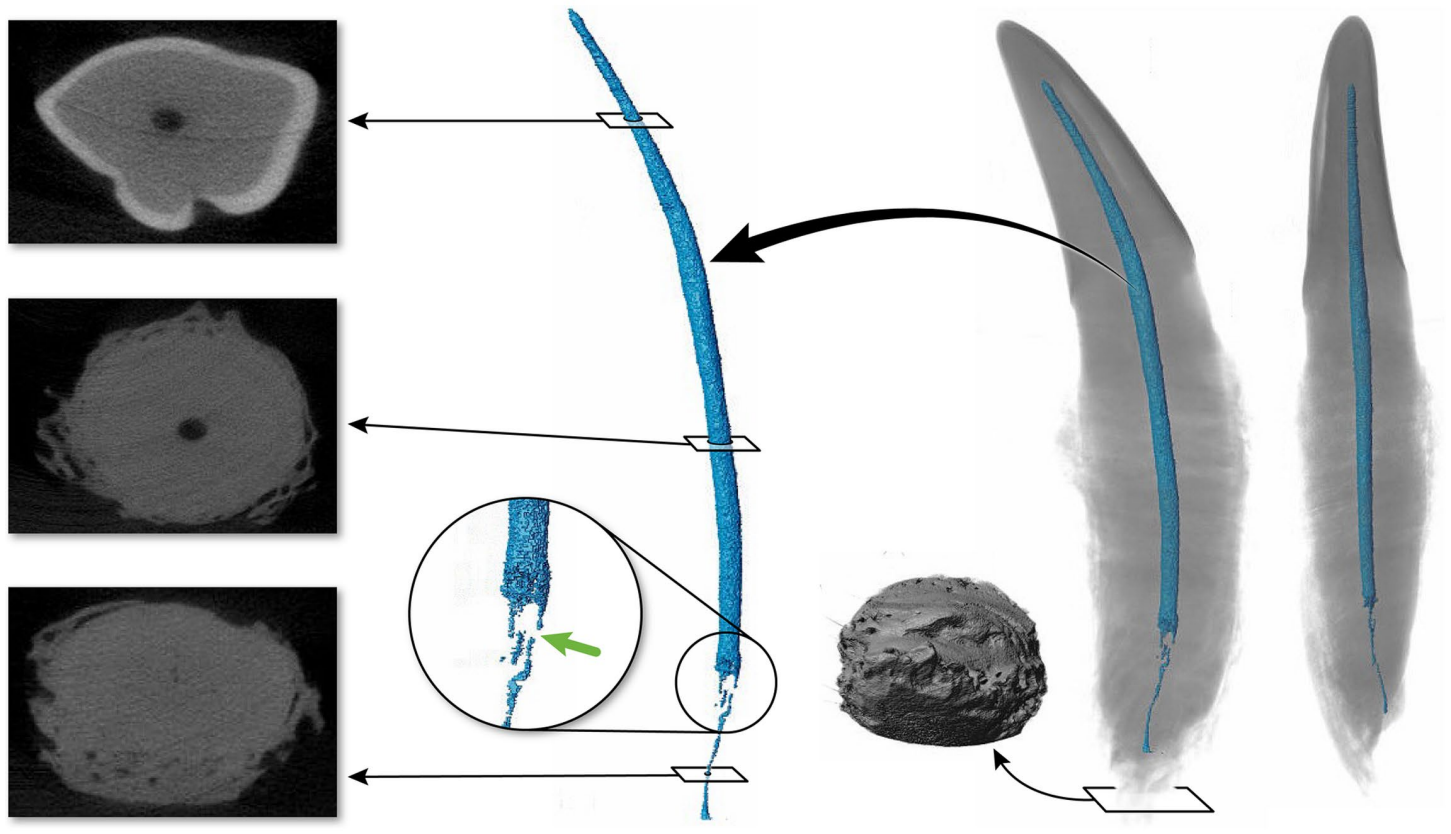
Maxillary tooth (36) affected by suspected tooth resorption or hypercementosis. The transverse images show irregularities in the cementum in the apical third. The 3D reconstruction of the pulp cavity is magnified into the area depicting a discontinuation of the pulp cavity. To the left of the tooth apex, a 3D apical view of the tooth is depicted.

A total of 44 canine teeth were extracted from 11 cadaveric specimens. After extraction, five teeth were excluded due to complicated crown and root fractures. Of these specimens, 39 canine teeth from 11 cat heads underwent micro-CT scanning and pulp cavity segmentation. An additional eight canines from two cadaver heads were excluded from the statistical analysis due to suspected early pathological tooth resorption or hypercementosis and only their anatomical descriptions and images are included (Figure 1).

Continuous variables are summarized in Table 1. Compared to mandibular canine teeth, the pulp cavity of maxillary canine teeth was significantly larger and longer, and the cusp-to-tip length was significantly longer. The maximum number of foramina at the apical delta was not correlated to the volume of the pulp cavity (Spearman’s *p* = 0.2796, *p*-value = 0.2943); the length of the apical delta was not correlated to the length of the pulp cavity (Spearman’s *p* = 0.1147, *p*-value = 0.6723); and the maximum number of foramina at the apical delta was not correlated to the length of the pulp cavity (Spearman’s *p* = 0.3376, *p*-value = 0.2010). The apical delta length was negatively correlated to the volume of the pulp cavity (Spearman’s *p* = −0.5441, *p*-value = 0.0293).

**Table 1:** Summary of the data comparing the parameters assessed between maxillary and mandibular teeth.

		Location		
Variable		Mandible	Maxilla	*p*-value
Volume (mm^3^)	N	7.00	9.00	0.0071
	Mean	8.05	13.70	
	Std dev	3.91	6.97	
	Min	2.30	2.90	
	Max	13.43	25.40	
	Median	8.58	13.57	
	Interquartile range	6.82	10.02	
Apical delta length (mm)	N	7.00	9.00	0.0626
	Mean	1.08	0.89	
	Std dev	0.38	0.45	
	Min	0.50	0.27	
	Max	1.80	1.83	
	Median	1.08	0.89	
	Interquartile range	0.17	0.55	
Pulp cavity length (mm)	N	7.00	9.00	0.0017
	Mean	16.43	18.26	
	Std dev	1.62	1.20	
	Min	15.00	16.72	
	Max	18.56	20.65	
	Median	15.27	17.93	
	Interquartile range	3.03	1.34	
Maximum number of foramina at apical delta	N	7.00	9.00	0.6280
	Mean	7.29	8.33	
	Std dev	3.51	4.09	
	Min	4.50	1.00	
	Max	15.00	15.00	
	Median	6.00	8.00	
	Interquartile range	1.50	5.50	
Cusp-to-tip length (mm)	N	7.00	9.00	0.0313
	Mean	1.81	1.97	
	Std dev	0.21	0.16	
	Min	1.49	1.71	
	Max	2.09	2.22	
	Median	1.80	1.92	
	Interquartile range	0.34	0.26	

### Pulp cavity morphology

**Figure 2:**
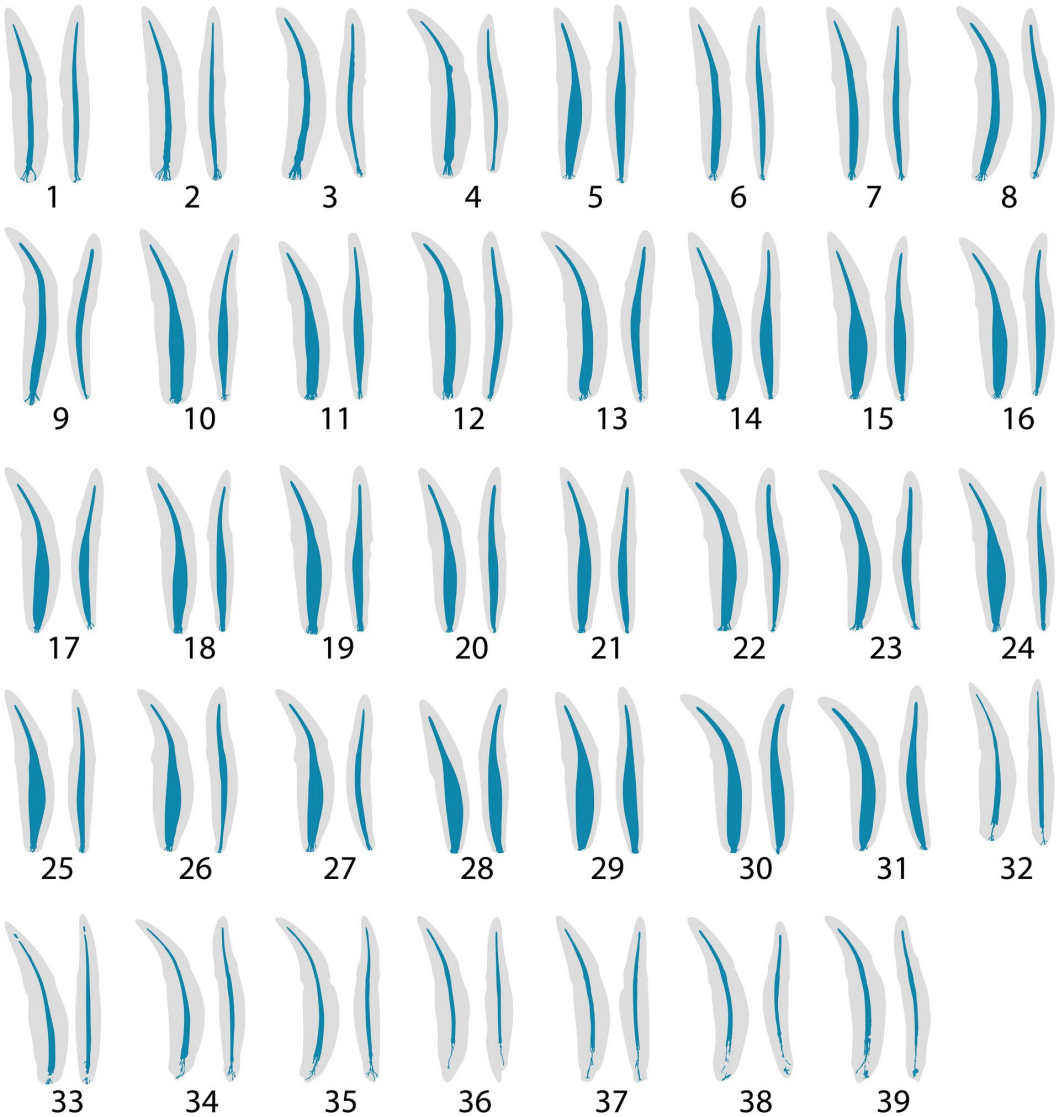
Buccal and mesial views of 3D reconstructions of thirty-nine maxillary and mandibular teeth. In each representative tooth, the left image depicts a buccal view, and the right image depicts a mesial view. Teeth 32–39 were excluded from statistical analysis.

**Figure 3:**
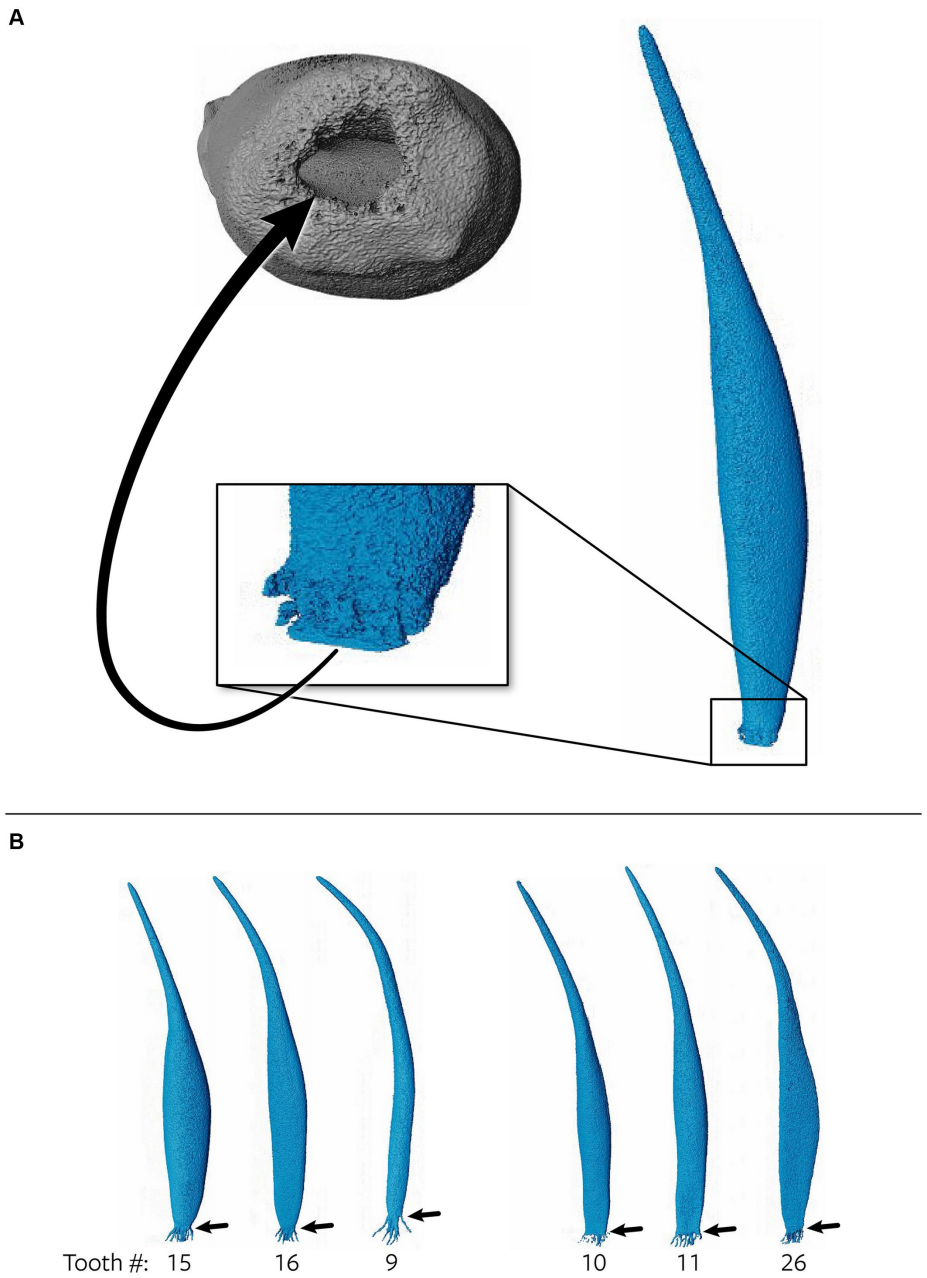
**(A)** Representative maxillary tooth (28) showing a single canal exiting from the apex as opposed to multiple foramina. The arrow points to an apical view of the single canal. **(B)** Teeth 15, 16 and 9 depict a constriction prior to the apical delta commencing, whereas teeth 10, 11, and 26 show no constriction prior to the apical delta.

**Figure 4:**
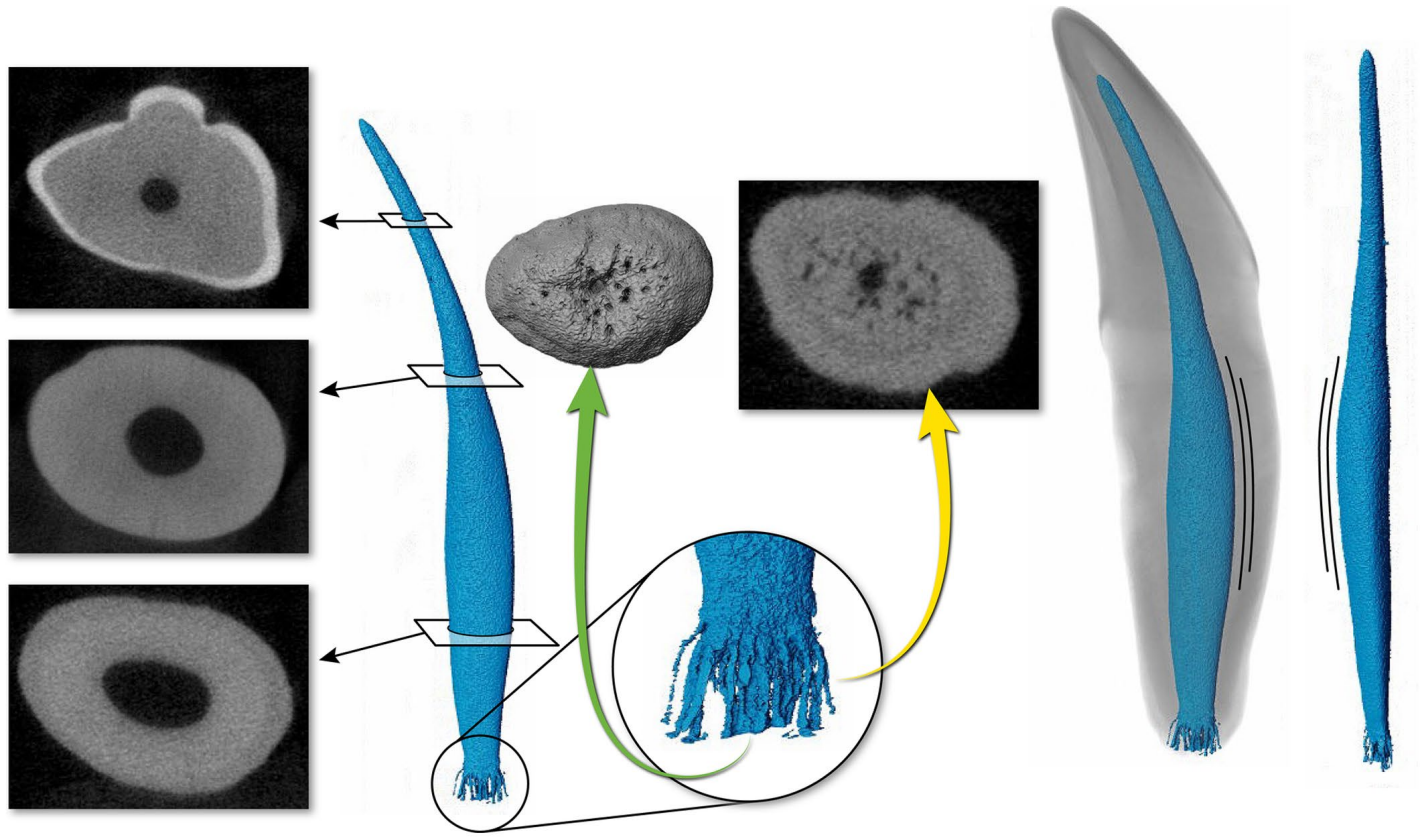
Representative maxillary canine (19) depicting a pulp cavity with a rounded, convex configuration paired with an oval pulp cavity in transverse cross section of the apical third. The green arrow demonstrates an apical view of the apical delta. The yellow arrow on the right depicts the apical delta in transverse cross section with the primary root canal being discernable.

**Figure 5:**
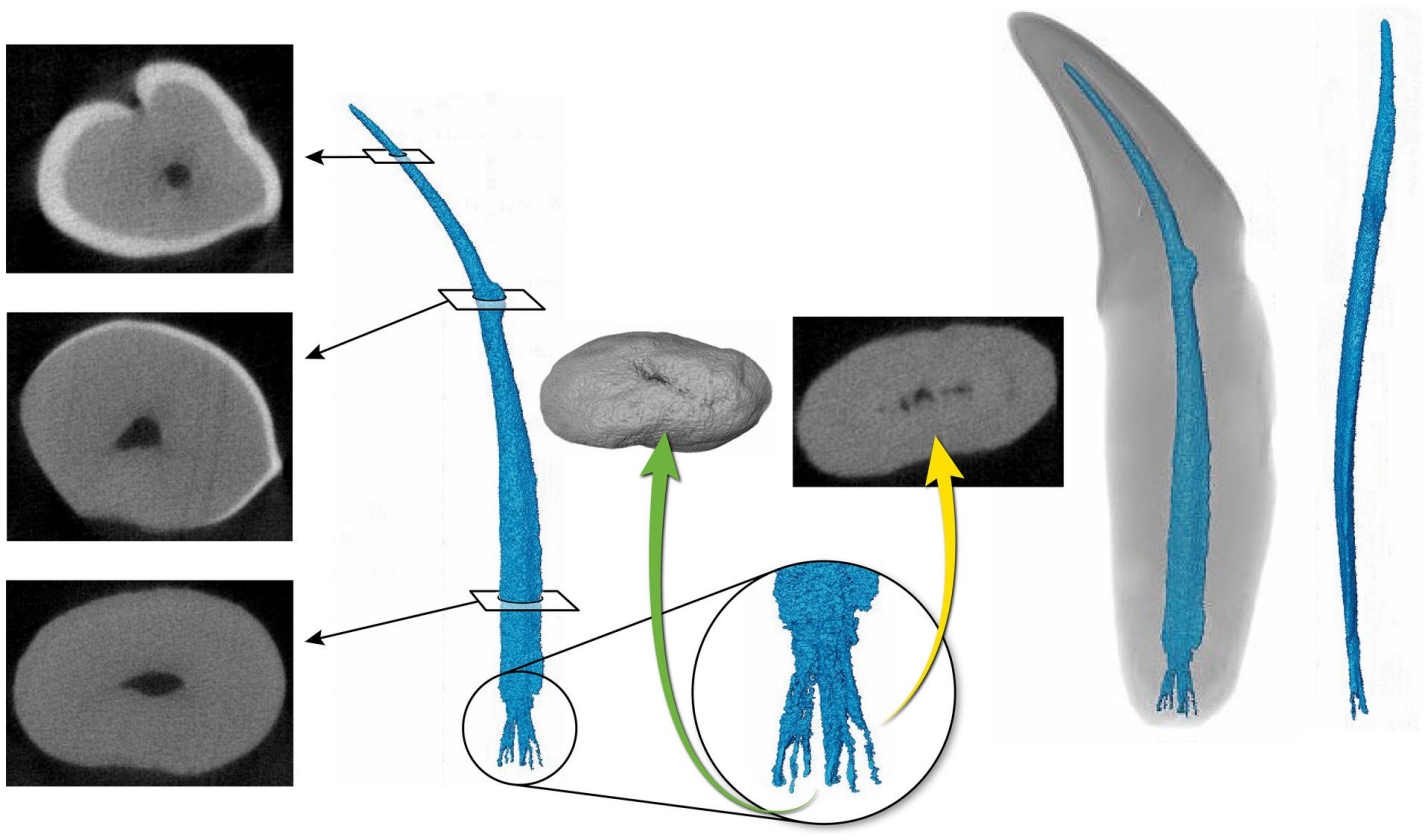
Representative mandibular canine (4) depicting a pulp cavity with a ribbon-like configuration paired with a flattened pulp cavity in transverse cross section. The green arrow is demonstrating an apical view of the apical delta. The yellow arrow depicts the apical delta in transverse cross section with the primary root canal being indiscernible.

The 3D reconstructions of the canine teeth showed that all specimens had one main pulp cavity (Figure 2). In every canine tooth, the pulp cavity extended from the most coronal point, down to the apex and exited through the most apical portion of the tooth. In 29/31 teeth, an apical delta was observed, whereas in 2/31 teeth, a single canal exiting out of the apex was noted (Figure 3A). A buccal view showed that most pulp cavities displayed an overall fusiform shape with the narrowest portion in the coronal third, widest portion at the middle third and leading to an apical narrowing (Figure 2). The eight canines with suspected early pathological tooth resorption or hypercementosis that were excluded from statistical analysis displayed a narrow coronal third with mild widening of the pulp cavity noted as it progressed to the middle third (Figure 1). However, when reaching the apical third, the pulp cavity became indiscernible with the surrounding dentin and appeared to contain calcifications, making it impossible to trace the entire pulp cavity down to the apex. At the junction of the root canal and the apical delta, 20/31 (65%) teeth depicted a constriction, whereas 11/31 (35%) did not (Figure 3B). The canals typically had a coronal curvature. Mesiodistal views depicted various configurations with 25/31 (81%) canals displaying a rounded convexity (Figure 4) and 6/31 (19%) canals depicting a ribbon-like flattened curvature (Figure 5). These ribbon-like pulp cavities depicted near linear configuration in transverse views (Figure 5). The 3D reconstructions showed that most pulp cavities exhibited a cylindrical shape in the coronal third, transitioning to an ovoid shape in the middle to apical third (Figure 4). The apical delta consisted of various configurations of an intricate system of multiple small canals dividing from the main root canal. In 15/31 (48%), the primary root canal within the apical delta could be clearly identified (Figure 4) and in 16/31 (52%) the primary root canal was indiscernible (Figure 5). No specimens (*n* = 39) were found to have lateral canals.

## Discussion

The 3D reconstructions of the pulp cavities presented in this study augments the minimal literature available describing the root canal morphology and anatomy of canine teeth in cats (5, 43). This study showed that the endodontic anatomy of canine teeth in domestic cats can vary considerably. The characterization of the anatomy noted in this study imparts valuable insight into enhancing the efficacy of root canal treatment in feline patients.

A significantly higher failure rate of root canal treatment has been recorded in cats as compared to their canine counterparts (34–36). Strom et al. (35) assessed the radiographic outcome of root canal treatment of 37 canine teeth in cats. The results showed that root canal treatment was successful or showed no evidence of failure in 81% of treated teeth and failed in 19%. In contrast, 127 teeth were evaluated after root canal treatment in dogs which found that treatment was successful or showed no evidence of failure in 95% and failed in 5% of teeth (36). It has been demonstrated that certain anatomical characteristics of the endodontic system can pose inherent challenges in achieving thorough debridement and sterilization during root canal treatment (1, 20, 38, 39). The 3D pulp cavity reconstructions presented in this study introduce insight into potential anatomic challenges that could be encountered during endodontic instrumentation.

The anatomical features of an apical delta may complicate the complete debridement of an infected root canal (9). The results of this study reveal that the apical delta morphology in feline teeth is consistent with a previous study describing a “sprinkler-rose” configuration (5). The apical delta configuration has also been shown in canine teeth of domestic dogs and ferrets using invasive techniques such as tooth clearing and staining for histopathology as well as using corrosive resin casts and scanning electron microscope (3, 4, 7, 44–46). However, further studies using non-invasive techniques for these species need to be established to define the complete anatomy of the canine tooth pulp cavity. In contrast to a previous study (5) which did not find an apical foramen in any specimens, the present study revealed two canine teeth that depicted a single canal exiting out of a single foramen (Figure 3A). Prior to analysis, all teeth were assessed radiographically for closed apices. These two teeth could be portraying an apex that was not fully formed or could reflect that some cats truly form an apical foramen. In addition, the apical delta length was negatively correlated to the volume of the pulp cavity, suggesting that in an immature tooth, the apical delta is shorter and not fully formed.

The thorough preparation and disinfection of the apical delta region is near impossible given the presence of sclerotic dentin, apical taper, and small diameter (47). Microorganisms in the apical portion of a root canal have been shown to play a significant role in endodontic treatment failures (48). Bacteria and debris may remain in recesses, unprepared walls, and apical delta canals, contributing to ongoing inflammation (2). Contrasting with the 13 ± 6 maxillary tooth and 12 ± 5 mandibular tooth apical ramifications reported in a previous study (5), a mean of 8.33 ± 4.09 and 7.29 ± 3.51 canals were noted in the maxillary and mandibular teeth, respectively, in the present study. This could have been a normal anatomical variation, or the complex apical anatomy may have resulted in an underestimation of the true number of canals in the apical delta. In comparison to the apical delta anatomy shown in dogs (3, 4, 44–46), it is possible that the canals of the delta in dogs are larger, and the use of irrigants results in more appropriate disinfection, contributing to higher success of root canal treatment. When considering the two species, it is also plausible that the apical delta plays a minor role in harboring bacteria, and it is not appropriate to extrapolate from human endodontic studies. Our results showed that the number of canals of the apical delta can easily reach >10 with various configurations, possibly lending to apical instrumentation challenges.

Pulp cavities are not homogenous and can have multiple different shapes in cross section at different levels of the same tooth (26, 49). Most pulp cavities in this study were narrowest in the coronal third with a circular cross section, then widened in the middle to apical third with an ovoid cross section (Figure 4). Out of the 31 teeth analyzed, six canines (19%) exhibited flattened ribbon-shaped canals (Figure 5). This ribbon-like shape likely results in some difficulty during shaping and debridement when using round rotary files (26). In contrast to the gradual taper commonly observed in human root canals (1), the opposite was seen in the 3D reconstructions of pulp cavities in the present study. This is a noteworthy difference as the endodontic instrumentation utilized in veterinary medicine is derived from human endodontic instruments. It is possible that the endodontic files being used are not appropriate for the thorough preparation and disinfection of the endodontic system in domestic cats. In addition, given the narrow coronal third of the pulp cavity observed, it is likely that a small endodontic file is selected during preparation, which is then not adequate for the wider middle to apical third of the pulp cavity. In wider pulp cavities, this may necessitate further enlargement of the access and excessive removal of coronal dentin to ensure adequate file size for shaping of the root canal.

The mechanical and biological objectives of root canal treatment in humans have been shown to be readily achievable in straight and large root canals (50, 51). Early attempts in human literature to classify root canal curvature suggested 4 main categories: straight or I-form, apically curved or J-form, entirely curved or C-form and multi-curved or S-form canals (37, 51). The 3D pulp cavity reconstructions in the present study showed significant variation in the degree of canal curvature. However, the pulp cavity curvature was not always consistent with the classification described in humans and no parallel could be obviously drawn and, therefore, curvature was not attempted to be classified. Despite that, variation in root canal curvature was observed that might be relevant for root canal treatment. Root canal curvature has been identified as a risk factor that may affect the outcome of endodontic treatment (37, 47, 50–52). A “J-form” curved canal has been identified as a moderate risk for endodontic failure and “C-form” and “S-form” as a high risk for endodontic failure (53, 54). Curved canals introduce factors that increase the risk of procedural errors and lead to persistent intracanal infection (37, 47, 52). Additionally, canal curvature reduces the cleaning efficacy of irrigation methods (37, 47, 52). In human studies, the most widely used method for measuring the root canal curvature magnitude is by using Schneider’s angle of curvature (37, 50). No attempts thus far have been made at classifying root canal morphology and curvature in veterinary species. The configuration of the pulp cavities in this study indicate that this may be a valuable area of future research to increase the success of root canal treatment in cats.

The quantitative evaluation showed that the maxillary canine teeth had a significantly larger and longer pulp cavity in comparison to mandibular canines (*p* < 0.05). The median length of the pulp cavity in mandibular and maxillary teeth was 15.27 mm (range 15.00–18.56 mm) and 17.93 mm (range 16.72–20.65 mm) respectively. This is different to previous findings (5), which showed that the anatomic root canal length of cleared mandibular and maxillary teeth was 11 ± 1.7 mm and 12.3 ± 1.6 mm. Although the total length of the pulp cavity of the teeth cannot be directly compared between studies, the results of both studies suggest that maxillary teeth are overall longer. This finding underscores the importance of treating maxillary and mandibular canine teeth as distinct entities and advises against approximating one to the other. A statistically significant difference of the cusp-to-tip length was found between maxillary and mandibular canine teeth (*p* = 0.031). This was expected as the maxillary teeth were both larger and longer than mandibular teeth. It is noteworthy that in the sampled teeth, 1.49–2.22 mm was necessary to reach the pulp cavity from the enamel tip. This gives clinicians a reference point for when to anticipate pulp exposure in fractures or during odontoplasty procedures.

Limitations of the present study were primarily associated with the small sample size and cadaveric *in-vitro* model. Given that the cadaver heads were commercially obtained, the heads were of unknown age, sex, and breed. These factors can influence the anatomy of the pulp cavity and, consequently, impact the endodontic treatment. Future studies should aim towards defining root canal anatomy of specimens with known signalment. Additionally, 44 teeth were anticipated for analysis, however, due to post-extraction processing, five teeth were lost due to complicated crown and root fractures. An additional eight teeth were excluded from the statistical analysis as early pathologic tooth resorption or hypercementosis was suspected on micro-CT, but was not evident on radiographic assessment (Figure 1). The suspected early pathology made the data collection difficult to obtain and likely inaccurate. Lastly, throughout the segmentation process, the presence of challenging anatomy, including dentin calcification, proximity of apical canals and artifacts, posed difficulty in accurately discerning canals of the apical delta. There was no statistically significant difference between the maximum number of foramina in the apical delta of maxillary and mandibular teeth. This could have been a normal anatomical variation, or the complex apical anatomy may have resulted in underestimation of the true number of canals in the apical delta.

In conclusion, we have characterized that the pulp cavity of maxillary canine teeth in cats is significantly larger and longer in comparison to mandibular teeth; that most canine teeth depicted apical deltas; and that pulp cavities showed varying curvature with the narrowest diameter with cylindrical configuration in the coronal third and the widest diameter with ovoid configuration in the middle to apical third. This study is the first step towards understanding the root canal system in domestic cats and provides insight into endodontic treatment considerations. Future studies are indicated to further understand the factors that contribute to success outcomes of endodontic treatment in domestic cats. Thorough understanding of the endodontic anatomy of the cat may impact the clinician’s choice in file systems and obturation materials and should be considered in future studies.

## Data availability statement

The original contributions presented in the study are included in the article/Supplementary material, further inquiries can be directed to the corresponding author.

## Ethics statement

The requirement of ethical approval was waived by Cornell University Veterinary Clinical Studies Committee (CUVCSC) for the studies involving animals because the experimentation was performed on cadaveric specimens and these procedures do not meet the description for use of animals in research, teaching or testing. The project was granted an exemption from IACUC Review (Protocol ID#: 011924-01). The studies were conducted in accordance with the local legislation and institutional requirements.

## Author contributions

EC: Data curation, Funding acquisition, Investigation, Methodology, Writing – original draft, Writing – review & editing, Conceptualization, Formal analysis. SP: Data curation, Formal analysis, Supervision, Validation, Writing – review & editing, Funding acquisition, Investigation, Methodology, Visualization. NF: Conceptualization, Methodology, Supervision, Validation, Visualization, Writing – review & editing, Funding acquisition, Investigation.

## Supplementary material

Supplementary Figure S1 Segmentation of the pulp cavity and 3D reconstruction of a representative maxillary tooth (19) on the Avizo software program. **(A)**, **(C)**, **(E)** are images in the “segmentation” window depicting the canine tooth in the XY, XZ, and YZ axis planes. **(A)** represents segmentation of the “top” of the pulp cavity where the blue colour highlights the course material created with the “Brush” tool, and the red colour highlights the fine material created with the “Magic Wand” tool. **(B)** represents the 3D reconstruction of the “top” pulp cavity. **(C)** represents segmentation of the “bottom” of the pulp cavity where the blue colour highlights the course material created with the “Brush” tool, and the red colour highlights the fine material created with the “Magic Wand” tool. **(D)** represents the 3D reconstruction of the “bottom” pulp cavity or the apical delta. **(E)** represents the “top” and “bottom” combined into a new material. **(F)** represents a detailed 3D reconstruction of the entire pulp cavity after manual refinements and the “Smooth” and “Fill” functions.
