# Supplementary figures and images for: Morphological study of pulp cavity anatomy of canine teeth in domestic cats using micro-computed tomography
Source: Front Vet Sci. 2024 Mar 8;11:1373517. doi: 10.3389/fvets.2024.1373517 (PMC10957770; doi:10.3389/fvets.2024.1373517)

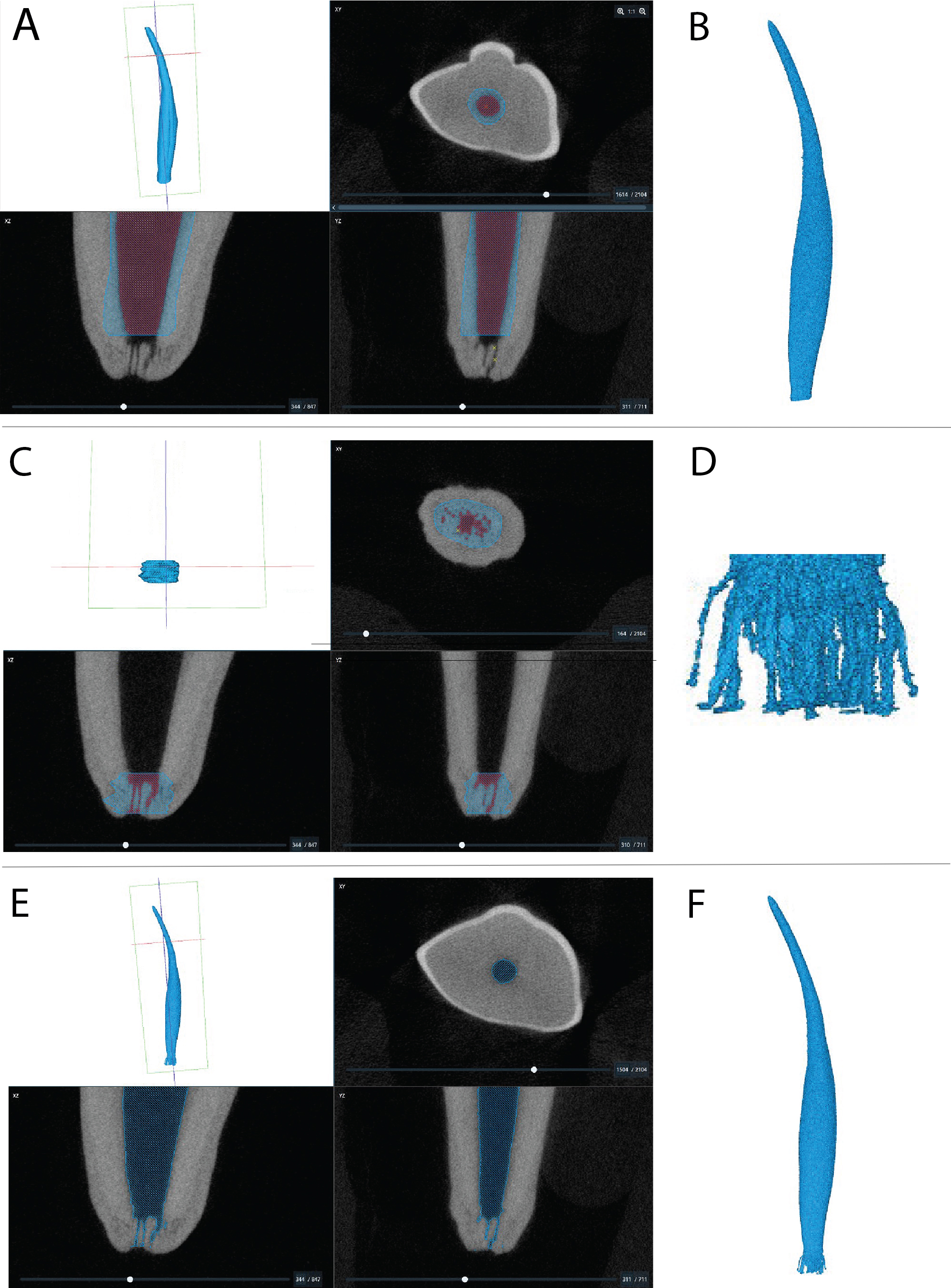

Supplement: Supplementary Figure S1 — Segmentation of the pulp cavity and 3D reconstruction of a representative maxillary tooth (19) on the Avizo software program. (A), (C), (E) are images in the “segmentation” window depicting the canine tooth in the XY, XZ, and YZ axis planes. (A) represents segmentation of the “top” of the pulp cavity where the blue colour highlights the course material created with the “Brush” tool, and the red colour highlights the fine material created with the “Magic Wand” tool. (B) represents the 3D reconstruction of the “top” pulp cavity. (C) represents segmentation of the “bottom” of the pulp cavity where the blue colour highlights the course material created with the “Brush” tool, and the red colour highlights the fine material created with the “Magic Wand” tool. (D) represents the 3D reconstruction of the “bottom” pulp cavity or the apical delta. (E) represents the “top” and “bottom” combined into a new material. (F) represents a detailed 3D reconstruction of the entire pulp cavity after manual refinements and the “Smooth” and “Fill” functions. [file Image_1.JPEG]
